# Ethical analysis of the change of values in healthcare

**DOI:** 10.1177/09697330251319374

**Published:** 2025-03-11

**Authors:** David Hansen, Silviya Aleksandrova-Yankulovska, Florian Steger

**Affiliations:** 9189 Ulm University, Germany

**Keywords:** Ethical analysis, healthcare, moral distress, moral injury, principlism, utilitarianism, value change, virtue ethics

## Abstract

What people value today can differ from what they have valued. But what does this value variability mean in the context of healthcare? We ethically analyze the current state of research on the change of embedded values in healthcare systems and the driving processes behind it. Starting with a systematic literature review and a content analysis, we subject the selected articles to an ethical analysis through three ethical theories: principlism, value ethics, and utilitarianism. The included papers demonstrated how moral dissonance between individual values and behavior leads to moral distress. The occurrence of moral distress was related to current healthcare practices. Beneficence and non-maleficence played a central role where principlism was considered, virtue ethics was criticized for not addressing the structural problems in the healthcare system, and consequences of value change for healthcare professionals and the society were analyzed. Further, principlism cannot fully cover the value change in medical care with its top-down and bottom-up processes leading to consequences for the patients, healthcare professionals, and society as a whole. We found correlations between top-down value change processes in the healthcare system and the quality of care. Health professionals are forced to develop an attitude that does not adhere to traditional medical values any longer and eventually leads to low-value care. Accompanying phenomena like moral distress cause dropout of healthcare workers. These can be hardly slowed down from the bottom-up by the development of resilience and moral courage. More effectively, structural changes through value interventions have the potential to improve working conditions and the quality of care.

## Introduction

People’s values can change over time. Throughout their lives, individuals develop and change their values. But what does this variability mean for healthcare?

Healthcare practice is closely linked to social and personal moral values.^1^ Ideally, individual values resonate with those of a care facility or healthcare system. Values represent what is important to people^2,3^ and serve as collective moral principles,^4^ motivating human behavior.^5^ They are often assigned to entities,^6^ which are seen as value bearers, and can be reflected in social structures. In that sense, we speak of the values that are embedded in care facilities or healthcare systems. However, they are not limited to moral values, that is, “right” and “wrong.”^7–9^ Beyond the sphere of medical ethics, other values, such as work efficiency, can also be relevant. Therefore, we aim to (1) ethically analyze and (2) critically discuss the current state of research on the change of embedded values in clinical practice and the healthcare system as well as to (3) describe the driving processes behind it.

It is consensus in moral psychology that human values have a universal structure and influence individual behavior based on their hierarchy.^10–12^ People naturally develop and rearrange values, giving them different priorities over time.^5,13–16^ In our article, “value variability” and “value change” refer to this adjustment within the value system. We hope our findings help facility managers avoid conflicts between professionals’ values and those of healthcare systems, and better prepare medical students for the moral challenges of their profession.

The concept of “value change” in modern healthcare systems has been explored, including the shift from moral-loaded to moral-free value sphere.^17,18^ One example is the prioritization of economic values over those associated with traditional quality care.

Value variability has been linked to conflicts between human values or challenging ethical issues.^19^ Healthcare professionals often respond to these conflicts with “coolout,”^17^ a form of moral desensitization, leading to a shift toward morally free value sphere. Since healthcare practice is filled with value conflicts and ethical decision-making, healthcare professionals are at a higher risk of moral distress than those in other fields.^19^

Moral distress is defined as a negative psychological reaction to morally challenging situations or to situations where a person knows what is right but is unable to act accordingly.^20–22^ Though often studied in nursing,^19,23,24^ all healthcare professionals can experience moral distress.^20,21,25–29^ It has even been described as an epidemic among physicians, hindering professionalism and empathy.^19^

Although often used interchangeably, moral injury differs from moral distress.^25,30^ Moral injury occurs with repeated exposure to moral distress^26^ and is defined as a lasting psychological wound, leading to anxiety, depression, and burnout.^25,30–32^ We suggest that “coolout” can be seen as an unconscious strategy to avoid moral injury by disregarding morals in morally distressing situations.

Value variability, that is, the potential to change over time, presumes also the existence of opposite processes helping healthcare professionals to improve their caregiving. This can be sparked by innovative teaching methods for best practices.^33–37^ Additionally, if healthcare management shares the same values as the staff, compliance is more likely.^38^ Over time, professionals build resilience, reducing moral distress and the risk of moral injury.^17^

## Methods

To establish a foundation for (1) ethical analysis and (2) critical discussion, we began with a systematic literature review. We, then, analyzed the selected articles using three ethical theories: principlism, value ethics, and utilitarianism, a method proven useful in similar studies.^39^ A narrative approach was chosen for the review due to the diverse methods used in the selected papers.^40,41^

**Figure 1.**
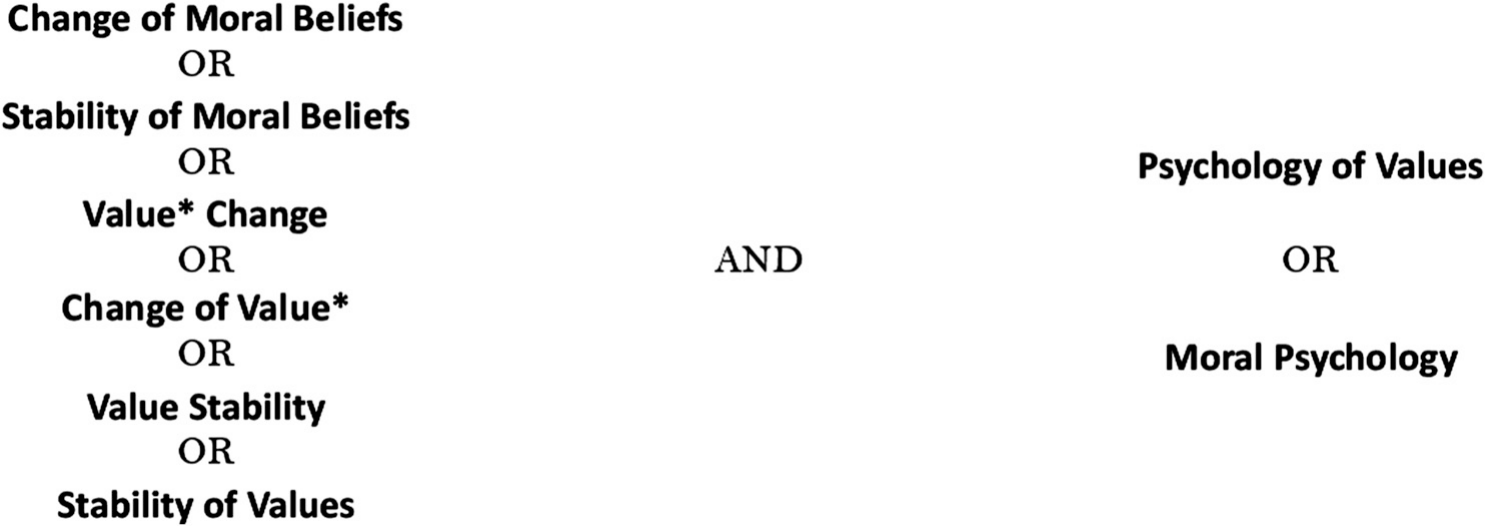
Keyword combination applied to the systematic literature search.

The search of literature was conducted in June–July 2023 on two databases, PubMed and Web of Science. To keep the results updated, we conducted a second search of the literature in April–May 2024. The search terms included “change of moral beliefs,” “stability of moral beliefs,” “value change,” “values change,” “change of value,” “change of values,” “value stability,” “stability of values,” “psychology of values,” and “moral psychology” (Figure 1). We added “moral beliefs” as it is often used synonymously with “Values.”^6,42^

**Figure 2.**
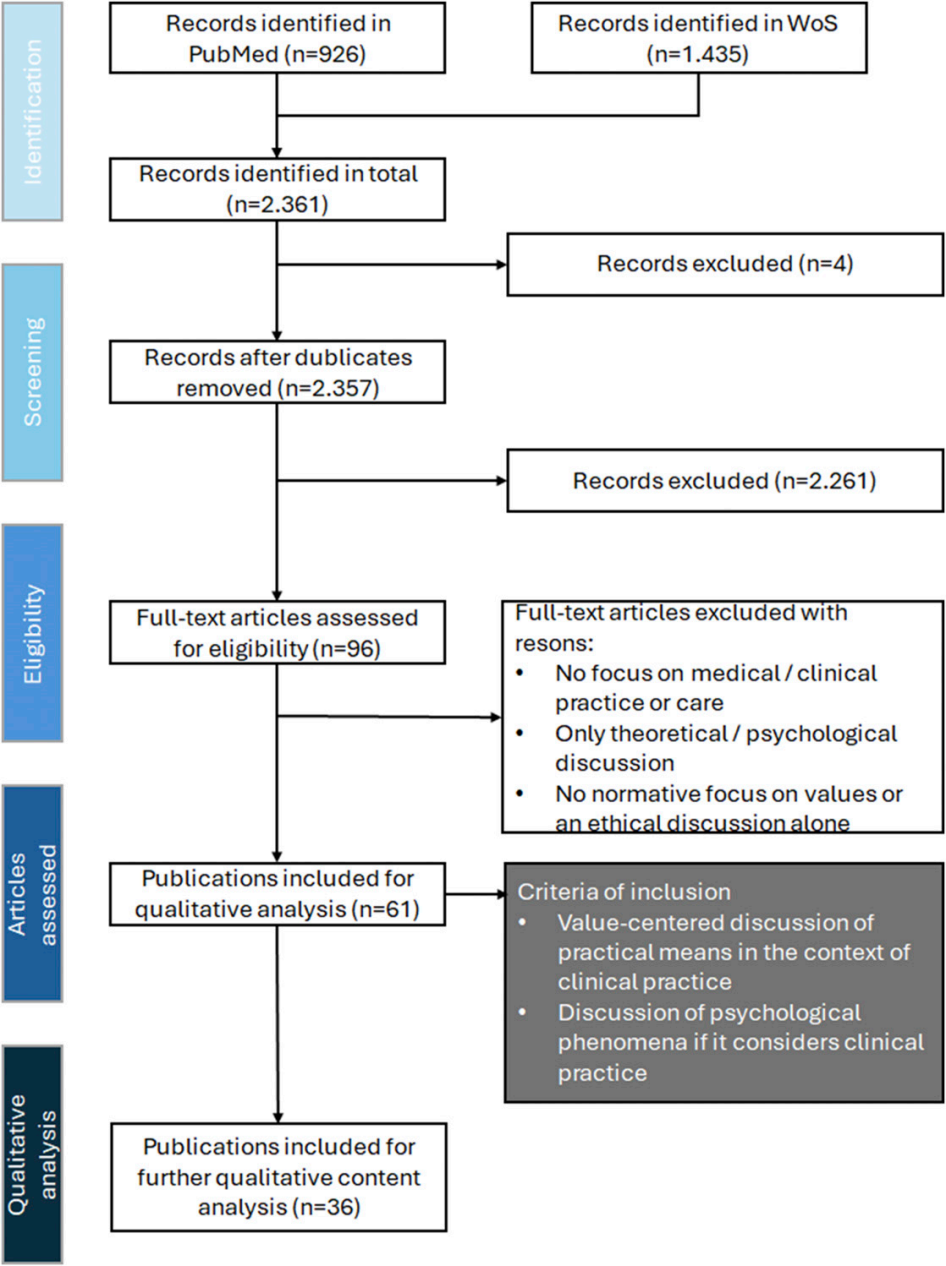
PRISMA flow diagram of the literature search process.

The search was limited to articles from the past 10 years to focus on the current healthcare situation, and Grey literature was excluded. We found 1435 articles on Web of Science and 926 on PubMed, including four duplicates. Based on titles and abstracts, 96 articles were deemed potentially relevant. Papers without reference to healthcare, normative values, or those focused on judgments, principles, or norms were excluded, leaving 61 for full-text analysis. Meta-ethical or abstract social-anthropological works were also excluded to focus on clinical practice. Finally, 36 articles were included for further analysis (Figure 2).

Publications meeting the inclusion criteria were thoroughly analyzed using content analysis.^43^ Themes were derived by comparing articles grouped into categories based on their thematic focus. The themes were then analyzed using the four bioethical principles: autonomy, beneficence, non-maleficence, and justice.^44^ This ethical analysis aimed to support a critical discussion of clinical practice, focusing on the relationships between healthcare professionals, their environment, patients, and relatives. However, since these principles were designed for individual therapeutic relationships, they proved insufficient for our system-level analysis. Therefore, we incorporated virtue ethics and utilitarianism to better explain the forces driving value change.

## Results

In total, we included six theoretical works,^19,29,45–48^ 14 reviews,^21,22,25,28,30,35,49–56^ four qualitative^5,26,33,57^ and three quantitative surveys,^20,38,58^ one case study,^27^ and nine randomized controlled trials.^34,37,59–64^ The thematic categories were, therefore, examined at different levels of evidence. We classified the articles through the prism of the chosen ethical theories, that is, principlism, virtue ethics, and utilitarianism (Table 1).

**Table 1.** Articles grouped according to the relevant ethical theory.

Ethical theory	Content	Reference to the literature
Principlism	Autonomy-related aspects	^28^
	Beneficence-related aspects	^47,48,58,59,62^
	Non-maleficence-related aspects	^47,58^
Virtue ethics	Values, attitudes, and character traits in general	^5,27,34,36,60^
	Moral courage	^22,35,45^
	Empathy	^29^
	Conscience	^50^
Utilitarianism	Consequences for healthcare system	^25,30,38,53,55,61^
	Consequences for healthcare staff	^21,37,38,46,54,56,63^
	Efficiency	^57,64^
	Cost effectiveness	^53^
	Lack of education	^19,20,26,49,52^

### Overview of the reviewed paper

One paper demonstrated how moral dissonance between values and behavior leads to moral distress.^19^ Other authors^29,45^ connected moral distress and burnout to current healthcare practices. The consequences of low-value care in the management of endometriosis in clinical practice^47^ and care for patients with dementia^58^ were discussed.

Effects of moral distress and moral injury were investigated from the different healthcare professionals’^21,22,25,28,50,53,55,56^ and undergraduates’^49,52^ perspectives. In one paper, moral distress was addressed for all professions^22^; other papers related the phenomenon to the affected doctors^21,29^ and nurses.^30,50^ In a survey among 939 Portuguese medical students, their preparedness for morally demanding work was studied.^20^ Further, a survey of 509 Pakistani workers in the healthcare sector looked for the relationship between organizational culture and mental health.^38^

One work derived conditions for the occurrence of moral injury and showed that there is still no uniform conceptualization in the literature regarding moral injury and moral distress.^25^ A connection to work relationships and values was described by one further work.^28^ Other authors distinguish between internal causes^22,35^ for moral distress, that is, value dissonances, and external causes, that is, systematic and structural problems in the organization of medical practice.^35^ Other studies underlined the importance of responsibility and the position in the clinical hierarchy for the occurrence of moral distress.^21,25,35^

Only in one study the social dimension in professional healthcare was discussed.^25^ It concludes that, despite the massive burden on the healthcare system, commitment on the side of the health professionals is still there and the system is dependent on it.

Different methods for learning value reflection through coaching and workshops^5,27^ were examined. In one focus group study, the authors presented 13 participants from psychiatric nursing with morally challenging situations in virtual reality by training software. An explicit correlation between moral distress and moral injury was found.^26^

Significant effects of value interventions like mindfulness training^34,36,37,59–61,63,64^ and interventions on the workplace acceptance^62^ were reported in several randomized controlled trials. However, one study discussed problems in gaining measurable effects.^63^

### Reading the included papers through the lens of the three chosen ethical theories

We identified **principlism** in six publications (Table 1). Only one publication^28^ dealt with conflicts between patients’ autonomy and beneficence. It explicitly stated that the tension between the autonomous will of the patient and the best possible care was a moral stressor. About one-third of healthcare professionals were involved directly in or witnessed actions violating the patient’s autonomy.^28^

The principles of beneficence and non-maleficence were central in many of the reviewed articles. In particular, we found two texts considering the effects of low-value care, that is, caring actions unlikely to provide any help,^46,56^ on patients with dementia^58^ and patients with endometriosis.^47^ Both cases show that low-value care is a particular burden to vulnerable patient groups. It reduces the beneficence of the treatment while causing maleficence. In the group of patients with dementia, the negative effects of low-value care affected 31% of all patients.^58^

Beneficence was found in publications dealing with moral distress as a current challenge to healthcare systems.^19,29,45^ Despite being known as related to the working environment,^55^ this phenomenon was targeted only in two of the studied articles. The interventions targeting moral distress alone were found to not affect the well-being of patients,^59,62^ despite that physicians perceived them as beneficial.^62^

We distinguished **virtue ethics** in 10 works where authors often referred to attitudes, norms, and values. Those are learned during interventions or higher education to guarantee professional and high-value practice.^29,34,35,45,60^ For example, one particularly important value for the healthcare professionals’ character is a positive attitude toward their job.^45^ Other examples of virtues were moral courage^35^ also called moral integrity,^22^ empathy,^29^ and conscience.^50^

Some authors explicitly criticize referring to these concepts as a solution to moral distress. The latter should be addressed through structural changes instead of leaving healthcare professionals alone coping with stressful situations.^45^

Other authors actively considered values as a source of drive and motivation^5^ and asked how and to what extent morality can be learned and taught.^36^ Physicians, for instance, might need professional help and coaching to activate reflection on values and apply it retrospectively to their practice.^27^

Finally, 20 publications referred to **utilitarian** concepts. The negative or positive effects of value interventions on the healthcare system or society were assessed and critically discussed.^52,57,61^ Two papers found a connection between the lack of integrated distress coping strategies and the increase in depression and anxiety among healthcare professionals^25,30^ particularly during the COVID-19 pandemic.^30^ Other authors focused on the cost efficiency of value interventions for strengthening healthcare professionals’ resilience instead of risking them dropping out of the system.^38,53^ It was observed that the implementation of such value interventions is obstructed on the side of healthcare managers, who don’t immediately see their economic value.^37,64^ Short mindfulness interventions were found to be not as efficient as expected.^51,54,63^

The consequences of education gaps for individual professionals were also addressed.^20,21,56^ At the undergraduate level, medical students felt themselves not sufficiently prepared for the stressful work environment.^20^ The implementation of value-based healthcare structures was found to have positive effects on the well-being of healthcare professionals.^56^ It was suggested that every individual should do daily exercises to build up resilience and increase overall personal well-being^46^ as well as to prepare for professionally challenging situations. The exercises could be done in a safe environment like virtual reality.^26^

## Discussion

### General overview

The included texts fall into two overlapping groups. The first focuses on how the working environment causes changes in values or value perception through moral distress, moral injury, or related concepts.^19–22,25,27,29,38,45^ Authors discussed clinical practice and explored strategies related to individual behavior,^30,46^ organizational structures,^61^ the working environment,^62^ and medical education at different levels.^36,49^

The second group consists of publications that describe,^47,58^ develop,^5,26,54^ and evaluate^26,27,34,37,57,61–64^ strategies for changing value perception or directing it towards a specific value set. Authors in this group considered interventions aiming to change existing clinical practices. The majority of publications dealing with mindfulness interventions^26,37,61,64^ reported positive results. However, in some cases, no effect was observed.^63^

According to our aim, these results shall now be (1) ethically analyzed through the three chosen ethical theories and (2) critically discussed. Finally, in the section “The meaning of embedded value change in healthcare”, we shall (3) describe the driving processes behind value changes in clinical practice.

### Analysis through principlism

It is a good clinical practice to make decisions together with the patient.^65–68^ This should lead to better outcomes for patients^69^ and directly impact their well-being. Therefore, this practice is eventually enhancing beneficence while reducing maleficence. But it is also time-consuming.^70^ Due to the current shortage of health professionals workforce,^45^ time is a limited resource. This is one practical example of situations where best clinical practice is not achievable. Instead, medical professionals are forced to deliver low-value care or they witness it as provided by their colleagues.^28^ As a result, the therapeutic decision-making process is affected and the standard of shared decision-making is undermined^65,66^ leading doctors to make paternalistic decisions^66^ which violates patients’ autonomy.

The change of embedded values in medical practice cannot be sufficiently studied through the four principles. Although they were applied to nursing ethics,^71^ the approach deals with the professional relationship with patients.^44^ Structural and organizational causes, mechanisms, and effects would not be covered. Some authors’ references to the influence of healthcare managers on the implementation of new practices are an illustration of this insufficiency.^57^ Also, principlism is not applicable where the effects are limited to the healthcare professionals’ well-being alone, causing moral distress and moral injury.^29,45,50,55^

### Analysis through virtue ethics and utilitarianism

Moral distress and moral injury cause drop-out of healthcare workers from the system.^18^ At the same time, these phenomena seem inherently to be part of the medical practice.^35^ It was, therefore, suggested to teach medical students and professionals resilience and moral courage.^22,35,45^ This is an example of strategies that aim to build up a virtuous character, thus preparing healthcare professionals for working in a morally challenging environment. In this regard, short-term effects were already reported in some intervention studies^34,61,62^ but still little is known about the long-term effects.^59^

It was also criticized that such solutions rather leave healthcare professionals to deal alone with a problem grounded at a structural level.^29^ Reducing the solution to individual exercises^46^ without proper accompanying changes in the working environment diminishes healthcare professionals to functional entities. Implementing these interventions into daily work would be more effective. Some authors see great discipline and virtuous character as a precondition for physicians’ well-being, thus preventing moral distress and moral injury.^46^ But this is a rather unrealistic picture of who is suited to be a professional. The healthcare system already suffers from limited human resources. The situation should not be exacerbated further by imposing unrealistic expectations on health professionals. However, single interventions also seem not to work.^57^ For instance, brief mindfulness training (30 min a week) alone leads to less significant results^51,63^ than holistic approaches, such as workplace acceptance and commitment therapy.^62^

Furthermore, it was observed that after enduring moral distress for a longer period, effects like “compassion fatigue” occur.^61^ This effect was also described as “coolout”^17^ indicating a state in which a clinical practice that contradicts the best-known care is not questioned any longer because of reaching a fatigue state. At first glance, this looks like a vicious character trait, that is, something that a good professional would not do. However, moral distress occurs exactly in those situations where the professional must act, and knows what the morally right action is, but cannot fulfill it.^45,50^ The action then was not done out of a “vicious character” but out of the circumstances.^72^

**Figure 3.**
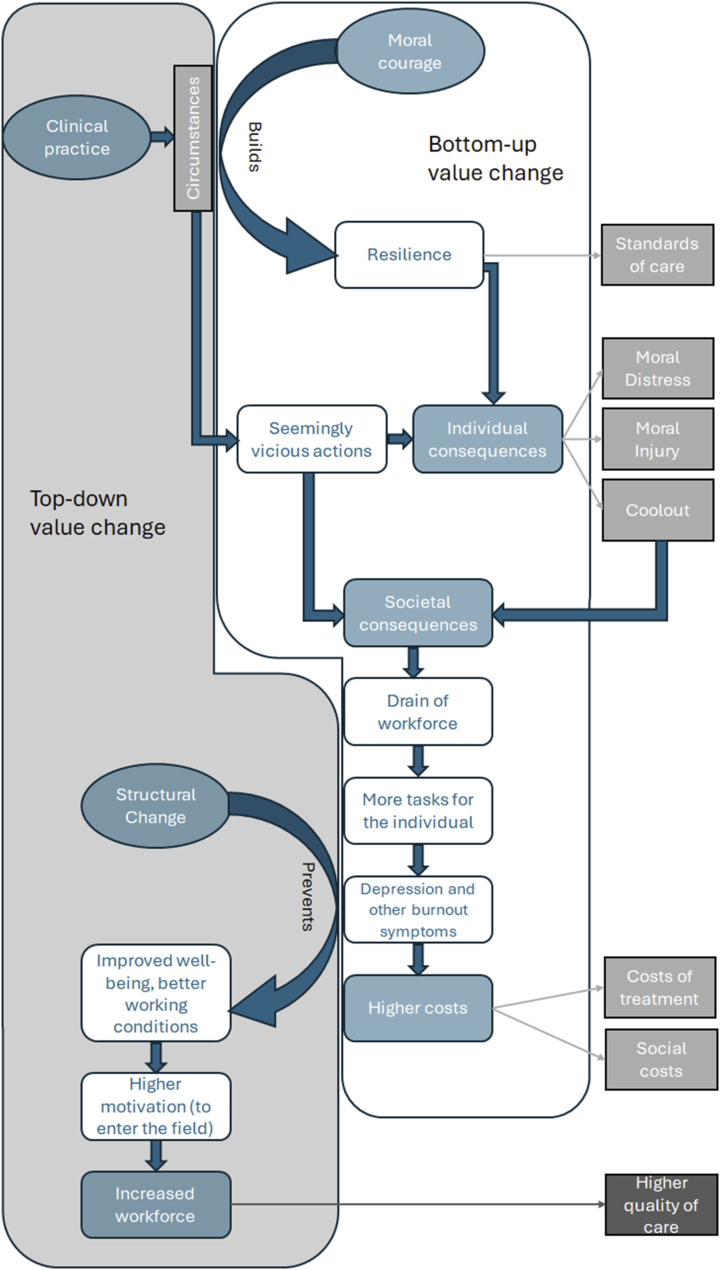
Value change processes overview.

From a utilitarian viewpoint,^73^ the imposed negative consequences are not limited to moral distress, moral injury, and coolout for healthcare professionals. These also extend to negative societal effects in the form of coolout-driven drain of workforce.^61^ The fewer health professionals work in the system, the more tasks the single healthcare professional takes over, the higher is the risk for burnout. The costs for the treatment of depression and other burnout symptoms of one healthcare professional were estimated to be two to three times higher than a single physician’s salary.^53^ Structural changes would prevent such a “vicious circle,” that is, self-reinforcing structures in healthcare that force healthcare professionals to act according to values they don’t believe in. This would also lead to increased well-being among physicians^38^ and better working conditions. It can be reasonably expected that this would further lead to an increase in the number of medical students^18,74^ and an improvement in the quality of healthcare on the patient’s side (Figure 3).

### The meaning of embedded value change in healthcare

Aiming to describe the driving forces behind value change in clinical practice, we found evidence for value change processes in two directions. As a top-down process, the value change from the ethical sphere to the sphere of economic values hinders the implementation of a structure that could be an effective instrument against moral distress and related phenomena.^57^ Healthcare professionals were forced to act according to embedded values they individually don’t believe in and suffer from individual consequences like moral distress, moral injury, and coolout.^17,61^

Additionally, this process implies consequences for the whole society (Figure 3). There are tensions between the values of best medical practice and the circumstances of clinical practice that force healthcare professionals to counteract the values of good medical care.^17^ Consequently, a drain of the workforce in the healthcare system and an increased workload for the remaining individuals occur.^18^ The risk of burnout increases for the remaining staff, and the whole society has to pay for the treatment of affected healthcare professionals and the costs of further shortages of healthcare resources.^48,53^

Several intervention studies showed how mindfulness training or value reflection can prevent burnout.^36,59,61,62^ The better the general working conditions are, the more the younger people are motivated to study medicine or nursing and enter medical professions. Eventually, this leads to a higher quality of care on the patient’s side.

Further studies are needed to optimize the implementation of such top-down processes into the working environment. The peer training model has not been successful.^63^ One idea is further professionalization in caring practices.^75^ Professional coaches, who are specially trained to take care of healthcare professionals’ mental health, could be integrated into the healthcare facilities to provide an ongoing service for the staff. Other ideas besides mindfulness training could involve virtual reality as an instrument to confront challenging situations in a safe environment.^26^ Role games have similar effects on the actors and the audience.^33^ Such forms of staff interaction might be of great help since the relations between different professionals are crucial sources of moral distress.^28^ Healthcare managers rather obstruct the implementation of structural changes^57^ because they typically don’t have close contact with other staff members, which makes them unable to see the practical value of structural changes. Interacting closely with the other staff may increase empathy for the other necessary roles in a healthcare facility and lead to a greater understanding of the individual professionals’ needs and the value of potential structural changes.

Since healthcare professionals in contrast to facility management^57^ have limited possibility to change the embedded values in their facility,^29^ reasonable doubts arise whether medical ethics training targeted only to clinical staff is effective. It was found that implementations were more successful if the management understood the logic behind the intervention. We showed that structural changes are beneficial not only to the healthcare professionals affected by moral distress, moral injury, and coolout but also to the whole society. Finally, it is also economically beneficial for the future healthcare system.

## Conclusion

This paper offers two findings. Firstly, we found two correlations between top-down structural processes with a potential for a value change to the ethical sphere of values and a higher quality of care. Value-based interventions were found to be particularly effective when the management was supporting the process. The involvement of different health-related professions and facility management was discussed as a potential way to increase the understanding of each other’s challenges in everyday life and, thus, increase the acceptance and stability of structural changes. Furthermore, moral distress, moral injury, and coolout were found to be a value-changing process that is directly correlated with increasing social and economic costs for healthcare services. Secondly, moral distress and related phenomena were caused by a working environment that forces medical professionals to counteract the values of best medical practice. This top-down value change process from the sphere of ethical values to a moral-free sphere of values is combated from bottom-up by the healthcare professionals’ individual development of resilience and a virtuous character of moral courage. We discussed this critically because healthcare professionals were left alone with structural problems that are not caused by them and affect their mental health with an increased risk of burnout. Thus, the development of resilience and moral courage would be more effective if combined with structural changes. The clinical practice, therefore, eventually will benefit from structural changes, value interventions, staff training, and the resilience of the healthcare professionals. However, facility managers should be involved in this process. In general, the participation of different staff seems to ensure a higher acceptance and stability of structural changes and increase the understanding of different challenges in the everyday lives of different professions.
